# Impact of chemical and physical treatments on the mechanical properties of poly(ε-caprolactone) fibers bundles for the anterior cruciate ligament reconstruction

**DOI:** 10.1371/journal.pone.0205722

**Published:** 2018-10-11

**Authors:** Amélie Leroux, Christophe Egles, Véronique Migonney

**Affiliations:** 1 Laboratory of Biomaterials and Polymers of Specialty, UMR CNRS 7244, Institut Galilée, Université Paris 13, Sorbonne Paris Cité, Villetaneuse, France; 2 Laboratory of Biomechanics and Bioengineering, UMR CNRS 7338, Sorbonne Universités, Université de Technologie de Compiègne, Compiègne, France; Institute of Materials Science, GERMANY

## Abstract

The anterior cruciate ligament rupture is one of the most common sport injuries. Due to ligaments’ poor healing capacity, surgical intervention is often required. Nowadays, these injuries are managed using replacement autografts or to a lesser extent using artificial ligaments. With the expansion of tissue engineering, more recent researches focus on the development of biodegradable structures that could allow graft functioning while enhancing host integration. The main challenge is to develop a structure that gradually loses its mechanical properties when at the same time the neo-ligament gains in solidity. Mechanical behavior and reconstruction of natural tissue are the two key points for such a successful device. This article evaluates the mechanical consistency of poly(ε-caprolactone) fibers bundles grafted with sodium polystyrene sulfonate, as a candidate for ligament prosthesis. In order to be medically used, PCL fibers need to cope with multiple steps before implantation including extensive washings, knitting, grafting and sterilization processes. The evolution of mechanical properties at each step of the elaboration process has been investigated. The results show that PCL bundles have the same visco-elastic behavior than the native ACL. Nevertheless, when undergoing physical treatments such as ionizing radiations, like UV or β-rays, the material endures a hardening, increasing its stiffness but also its fragility. At this opposite, the thermal radical grafting acts like an annealing step, increasing significantly the elasticity of the PCL fibers. With this chemical treatment, the stiffness is decreasing, leading to higher energy dissipation. Added to the observation of the structure of the material, this demonstrates the possibility of the PCL to modulate it microstructure. In case of orthopedic prosthesis, the need of such a construct is strongly required to avoid distension of the future prosthesis and to restore good knee stabilization, showing the promising future of PCL ligament prosthesis.

## Introduction

The anterior cruciate ligament (ACL) rupture is a common injury which mainly affects young and active population[1]. Faced to this problem, the development of synthetic devices for ligament reconstruction began in the early 80’s[2]. After a multiple range of non-adapted materials used, leading to synovitis[3–5] or prosthesis rupture[6,7], the choice of poly(ethylene terephthalate) (PET) appeared to be promising[8–10]. Nevertheless, nowadays the non-degradable PET synthetic ligaments commercialized still encounter limitations such as the anchorage to the bone, the release of tear particles due to abrasion and a poor cell recolonization with limited tissue ingrowth[11]. Given that, many studies are conducted with the purpose of finding a material and a design which can have similar mechanical properties than the native ACL and be well integrated in the body avoiding adverse reactions as foreign body response [12–15]. Different kinds of surface treatments are possible to improve the bio-integration of prostheses, such as the control of the surface roughness, surface chemistry to increase the hydrophilicity[16] or the grafting of bioactive molecules as peptides or drugs [17–19] able to interact positively with the biological environment[20]. Moreover, with the expansion of tissue engineering applications, the last objective is to develop biodegradable ligament prostheses which degradation rate should guarantee resistance to mechanical stress during the required time to the formation of a regenerated ligament and can act as a scaffold for cells colonization[21–23].

Our laboratory has been working for several decades on the elaboration of bioactive surfaces by using sodium poly(styrene sulfonate) (pNaSS) functionalization to allow the control of the cell response[24,25]. pNaSS polymer has been covalently grafted on PET LARS ligament by using a grafting “from” technique in order to improve the cell response[26,27]. After promising *in vitro* studies results [26,28], an *in vivo* study on sheep confirmed good recovery after 3 and 12 months implantation, demonstrating an improvement of the cell adhesion and colonization along the PET fibers of the prosthesis as well as the cell activity which can be compared to that of native ACL [28,29]. With the knowledge of this surface chemistry control and within the tissue engineering context, we started working with polycaprolactone (PCL) as a candidate for the elaboration of a future degradable synthetic ligament. PCL is a semi-crystalline polyester which has its glass transition temperature (T_g_) at -60°C and its melting point in the range of 59°C to 64°C[30] and which degradation is estimated to take 3 to 4 years depending on its molecular weight[31–33]. It exhibits a viscoelastic comportment[34], presuming being a good material for ligament reconstruction. In addition, PCL is already used in medical devices on the market such as suture, ophthalmic patch graft, intrauterine device etc., so that its biocompatibility is well-established[30]. The authors have first developed the pNaSS grafting process on PCL films[35,36] and the process extended to PCL fibers bundles is presented in this study.

In order to achieve and control the extrusion of polymer fibers, the textile industry uses spin finish, waxes or lubricants [37]. Unfortunately, these manufacturing products can be harmful when released from implants in body fluids of patients. Then, in order to be medically used, PCL fibers need to cope with multiple steps before implantation including extensive washings to remove spin finish, knitting into a final prosthesis, grafting process to covalently bind bioactive pNaSS and finally, sterilization process. In this article, we investigated the mechanical properties of PCL fibers bundles after the different steps of the elaboration process: crude fibers, fibers after spin finish removal, fibers that have undergone the pNaSS-grafting process and sterilization. After the elaboration and functionalization of the samples, we firstly checked the presence of the pNaSS grafting using a colorimetric assay. Then, the samples were seeded with primary sheep ACL cells in order to confirm the bioactive feature of pNaSS grafted-PCL fibers. In parallel, we tested the mechanical properties of PCL fibers through different strain rate to define optimal conditions of mechanical assays. Afterwards, we applied an axial load until samples rupture and determined the intrinsic mechanical properties. Loading experiments were achieved to validate the limit of the elastic domain. Finally, in order to understand the mechanical behavior of the pNaSS grafted and ungrafted PCL bundles, we correlated the mechanical properties results to the microstructure of PCL films elaborated following different thermal treatments.

## Material and methods

### Samples preparation

#### Preparation of PCL fibers bundles

20 PCL fibers of 110±15μm diameter each (Purasorb PC 12, Luxilon Industries—Belgium) are associated and tied together in order to form 40±10 mm length fibers bundles. To remove the spin finish, bundles 10% (w/v)) are sequentially ultrasound washed as following: hexane for 15 min, absolute ethanol for 5 min, then water two times for 5 min. Samples are then dried under vacuum and stored at 4°C until grafting process or experiments.

#### Preparation of PCL films

Spin coating. PCL films are manufactured using a spin-coating method. A PCL (Sigma-Aldrich, Mn = 80 kDa) solution in dichloromethane (30% (w/v)) is dropped onto a glass slide and then spun for 30 seconds at 1500 rpm using a SPIN150-v3 SPS. Films are then dried overnight at room temperature to evaporate the solvent.

Thermal treatments. The following thermal treatments are achieved on PCL films: (a) 4 days at 45°C, (b) 10 min at 60°C (melting temperature) then tempered in frozen water. Films are then cut into small disks of 14 mm ± 1 mm diameter and stored at 4°C before observation in SEM. SEM images were carried out using a Hitachi TM3000 SEM operating at 15 kV.

### Grafting of poly(sodium styrene sulfonate (pNaSS) on PCL bundles

PCL bundles samples are functionalized with polyNaSS using a grafting “from” technique. After the spin finish removal step, PCL bundles are ozonated at 30°C in distilled water under stirring for 10 min. Ozone is generated using an ozone generator BMT 802 N (ACW) with a gas pressure of 0.5 bars and an oxygen flow rate of 0.6 L min^-1^. Secondly, the ozonated PCL samples are transferred into a degassed aqueous NaSS solution (15% (w/v)) under argon and maintained either (i) 3 hours at 45°C under stirring for thermal grafting, or (ii) 30 min under UV (irradiance 10 W/cm^2^, Omnicure Serial 2000, Polydispensing system) for UV grafting, in order to allow the radical polymerization of the monomer. Then, samples are extensively washed with distilled water for 48 hours and then vacuum-dried. The evidence of the pNaSS grafting is provided by toluidine blue colorimetric assay and the grafting rate is determined according to Ciobanu et al [38].

### Cell culture

#### Cell isolation

Anterior cruciate ligaments (ACL) from one sheep (a 2-year-old female pré-Alpes sheep, weighing approximately 60 kg, free of degenerative joint disease) were donated from a tissue back thanks to the collaboration with Pr V. Viateau at the ENVA, Maisons-Alfort, France (Ligart protocol). Tissues are cut into small pieces of 1 to 2 mm^2^, washed three times in DPBS (Gibco) and incubated in a 0.1% (w/v) collagenase (Sigma-Aldrich) solution for 6 hours at 37°C under 5% CO_2_. The mixture solution is centrifuged (3 min at 1500 rpm): the supernatant is withdrawn and the clot is resuspended in DMEM (Gibco) complemented with 10% Bovine Calf Serum (Sigma-Aldrich), 1% Penicillin-streptomycin (Gibco), 1% L-glutamine (Gibco); cells are cultured in flask until confluence and prior seeding on samples.

#### *In vitro* study

pNaSS-grafted and ungrafted PCL bundles samples are placed on the bottom of a 24-wells plate using Teflon inserts. Primary sheep ACL fibroblasts (sACL) are seeded onto the PCL fibers bundles at a density of 5.10^4^ cells/well and cultured 3 days at 37°C, 5% CO_2_. After this period of time, the bundles are observed with an inverted phase-contrast microscope (CKX31—Olympus).

### Sterilization

Thermally pNaSS grafted fibers bundles were sent to LARS Company for undergoing beta sterilization according to the authorized final process currently used for PET ligaments sterilization.

### Mechanical testing

All the mechanical assessments are carried out on PCL bundles samples by using a Bose Electroforce 3230 equipment (Bose).

#### Traction experiments

Differently treated PCL bundles (effective length = 10mm) have undergone with tensile loading until rupture. Stress strain curves until rupture are recorded and Young’s Modulus E in MPa, elongation ε in % and the ultimate tensile stress (UTS) in MPa are determined.

#### Load and unload experiments

Load-unload and fatigue fracture tests. Stress fracture is studied by alternatively applying load and rest periods to the PCL bundle samples. The different bundles samples are submitted to the following loads 3.5, 24 or 51N. The consecutive dissipation of energy is calculated as the integral of the area enclosed by the hysteresis curve from the load displacement graphs.

### Statistical analysis and software’s

All the experiments are carried out a minimum of 8 times to get at least 6 usable results. Statistics are calculated with ANOVA test (p≤0,005 is considered statistically significant). Lengths of cells and spherulites are measured with Image J software. Energies dissipation are calculated with OriginPro 8 software.

## Results

The presence of grafted pNaSS molecules on PCL fibers bundles samples was determined according to the toluidine blue assays process. The obtained average value of the grafting rate of the fibers bundles (n = 20) equals 1.14 ±0.50 μmol.g^-1^.

### Cell culture *in vitro* assays

**Fig 1:**
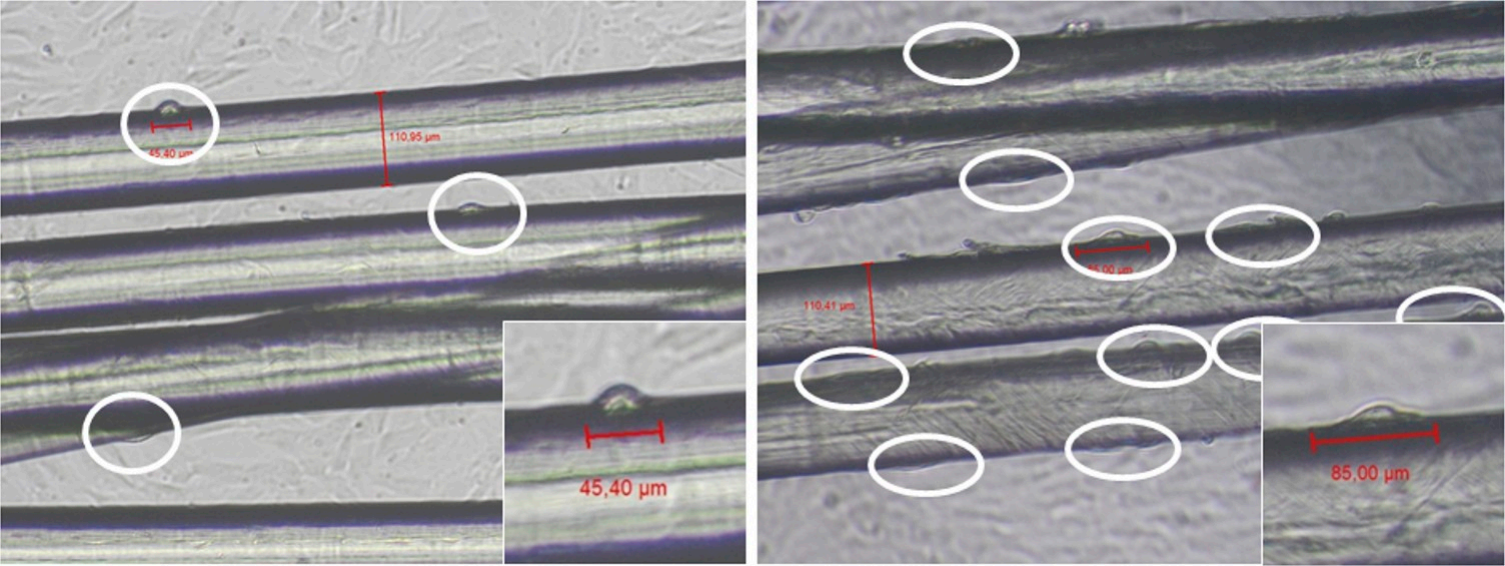
**Images of primary sheep ACL cells seeded onto ungrafted (left) and grafted (right) PCL fibers bundles after 3 days of culture**.

After 3 days of culture, samples were observed with an inverted microscope and several pictures of the alive culture were taken. Cell lengths were measured using Image-J software. The review of all pictures pointed out that the cell morphology along the PCL fibers of the bundles was clearly different if cells were seeded on pNaSS grafted or on ungrafted fibers bundles as shown in Fig 1. It was observed that the cells were longer and in line with the fibers when they were on the grafted fibers. The mean length of the cells was found to equal 49.5±17 μm on the ungrafted samples whereas it equals 76.5±27.7 μm on the grafted samples. Results showed that cells are two times more spread (+ 54%) when PCL fibers were grafted by pNaSS in comparison to virgin PCL fibers. In addition, the number of adherent cells on surfaces is also higher on the pNaSS grafted- fibers bundle when compared to the ungrafted one as shown in Fig 1.

### Mechanical properties

#### Determination of the optimal strain rate conditions

Different strain rates were tested to determine the optimal conditions of the mechanical assays on PCL fibers bundles (Table 1). Results presented in Tables 1 and 2 evidenced the variations of the deformation when varying the strain rate. According to these results, the best condition which corresponds to the lower value of the strain rate equals 3.6 mm.min^-1^ and corresponds to 0.6% sample deformation per second. Indeed, at this strain rate the slippage of the fibers inside the grips is limited and allows sufficient observation time before rupture, with an average of 210 seconds before the sample break. This strain rate value was then used for all the following mechanical experiments.

**Table 1:** Experiment times and percentages of sample deformation per second at different strain rates tested.

strain rate (mm.min^-1^)	3.6	6	10	30	50
**Time of experiment (sec)**	210	126	76	26	15
**Percentage of deformation/sec**	0.6%	1%	1.7%	5%	8.3%

**Table 2:** Mechanical data obtained from experimental curves.

	Young’s modulus	Elastic limit	Yield stress	Elastic strain	Maximum load	Ultimate Tensile Stress	Ultimate strain
	MPa	MPa	MPa	%	N	MPa	%
**Bundles with strain rate of 0.06 mm/s**	1400 ± 92	163.05 ± 7.99	-	13.65 ± 1.00	59.58 ± 1.25	313.49 ± 6.57	81.96 ± 3.22
**Bundles with strain rate of 0.10 mm/s**	1482 ± 39	158.33 ± 0.61	-	12.10 ± 0.17	59.21 ± 1.68	311.51 ± 8.84	83.84 ± 5.38
**Bundles with strain rate of 0.17 mm/s**	1398 ± 96	168.56 ± 10.04	-	13.05 ± 1.14	59.67 ± 1.86	313.92 ± 9.79	78.86 ± 4.61
**Bundles with strain rate of 0.50 mm/s**	1506 ± 71	170.45 ± 8.98	-	12.79 ± 0.58	61.29 ± 0.71	322.47 ± 3.72	84.54 ± 2.86
**Bundles with strain rate of 0.83 mm/s**	1547 ± 60	179.44 ± 11.24	-	13.36 ± 1.32	-	-	-
**Crude bundles**	1400 ± 92	163.05 ± 7.99	246.69 ± 7.94	13.65 ± 1.00	59.58 ± 1.25	313.49 ± 6.57	81.96 ± 3.22
**Bundles without spin finish**	1404 ± 110	159.36 ± 5.10	246.3 ± 5	13.21 ± 0.40	58.63 ± 2.62	308.47 ± 13.78	76 ± 6.8
**Bundles after ozonation**	1324 ± 45	160.20 ± 6.60	249.41 ± 2.03	16.06 ± 0.79	59.34 ± 1.42	312.20 ± 7.45	91.08 ± 5.64
**Bundles after thermal treatment without pNaSS**	1098 ± 82	150.20 ± 6.49	239.36 ± 8.12	18.67 ± 1.05	55.74 ± 2.20	293.25 ± 11.56	92.62 ± 8.33
**Bundles after thermal grafting**	1125 ± 49	161.42 ± 11.24	237 ± 11	18.80 ± 1.32	53.21 ± 2.67	279.98 ± 14.06	74.88 ± 10.84
**Bundles after UV grafting**	1301 ± 49	133.30 ± 10.28	226 ± 7	12.65 ± 0.97	52.60 ± 1.40	276.74 ± 7.36	77.89 ± 1.78
**Bundles after thermal grafting + β sterilization**	1258 ± 51	126.05 ± 8.91	199.50 ± 3.35	15.40 ± 1.40	41.40 ± 0.61	217.80 ± 3.23	78.50 ± 4.23

#### Influence of chemical and physical treatments on the mechanical characteristics of the pNaSS grafted and ungrafted PCL bundles

**Fig 2:**
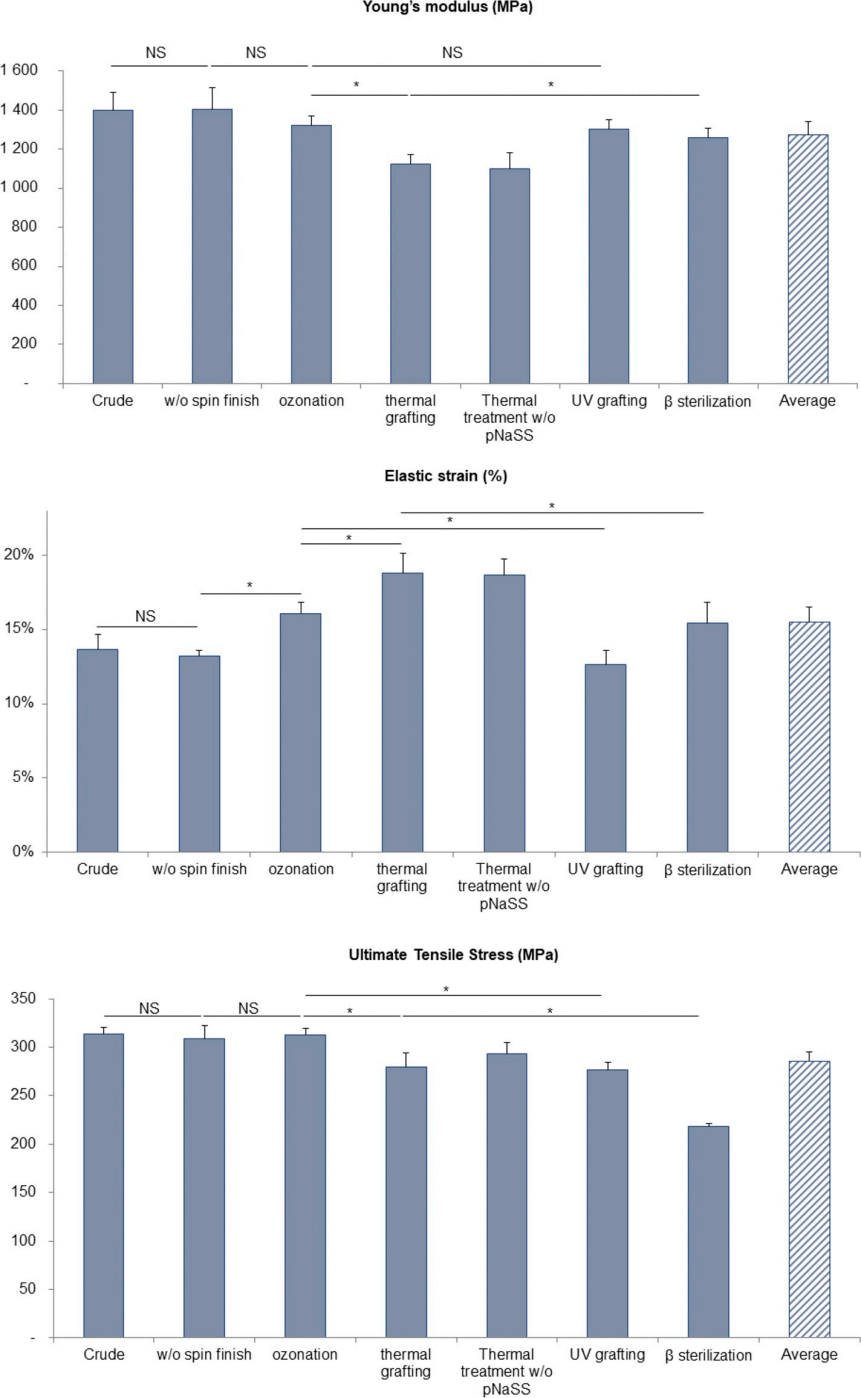
Young’s modulus, elastic strain and ultimate tensile stress obtained from stress-strain curve after different chemical and physical treatments (NS = Not Significant / *p≤0,005).

The mechanical characteristics—Young’s modulus (E), elastic strain (ε) and ultimate tensile stress (UTS)—were determined for PCL bundles having undergone different treatments–spin finish removal, pNaSS grafting comprising ozonation, polymerization at 45°C or under UV and sterilization–(Table 2 and Fig 2).

**Crude fibers and spin finish removal step:** Crude PCL fibers bundles: the Young’s modulus E equals 1400 ± 92 MPa, with an elasticity ε value of 13.65 ± 1.0% and a UTS of 313.49 ± 6.57 MPa.

Removal of spin finish: The three values E, ε and UTC are quite identical before and after the removal process of the spin finish (see Table 2).

**pNaSS grafting process:** The grafting of pNaSS is carried out in two steps: (a) an activation step of ozonolysis to create hydroperoxide and peroxide groups on the PCL surface (oxidation), (b) the polymerization of NaSS from the activated surface (radicals’ generation and radical polymerization). The mechanical characteristics of the samples after each step were determined and showed that:

- the first step of ozonation doesn’t impact neither the Young’s modulus E or the ultimate tensile stress UTS values whereas the elasticity is significantly increased (21%) varying from 13.21 ± 0.40% to 16.06 ± 0.79%
- the second step–polymerization from the surface by thermal grafting–is achieved in water for 3 hours at 45°C. This step is evidenced to significantly impact all the mechanical characteristics: the Young’s modulus and the ultimate tensile stress are both decreased, 17% for E which varies from 1324 ± 45 to 1098 ± 82 MPa and 6% for UTS which is decreased from 312.20 ± 7.45 to 293.25 ± 11.56 MPa while the elastic strain is 16% increased from 16.06 ± 0.79 to 18.67 ± 1.05%. These tendencies were identically observed when fibers bundles have undergone the thermal grafting step of 3h at 45°C but without the presence of the NaSS monomer in the solution, demonstrating that these modifications are linked to the temperature treatment and not to the presence of the monomer.
- the UV grafting process, does not impact the Young’s modulus as observed after the ozonation step. A slight decrease (11.4%) of the ultimate tensile stress is observed, UTS varies from 312.20 ± 7.45 to 276.74 ± 7.36 MPa. Interestingly, we observed a 26% decrease of the elasticity which varies from 16.06 ± 0.79 to 12.65 ± 0.97%, going back to the ε observed value of the fibers after the removal of the spin finish. Taking this into account, the previous augmentation of the elasticity observed after the thermal grafting is not linked and cannot be attributed to the covalent binding of pNaSS.

**Sterilization impact:** The sterilization process was carried out by β rays. It has been achieved on the thermal grafted bundles. Results in terms of mechanical characteristics showed that the β rays sterilization process induced: (a) an increase of the samples stiffness when comparing the Young’s modulus value to that was observed after the thermal grafting step (1125 ± 49 MPa); indeed, E value recover the observed value after the ozonation step and equals 1258 ± 51 MPa, (b) a decrease of the elastic strain reaching ε value previously obtained after the ozonation step and equals to 15.40 ± 1.40%, (c) a significant decrease of the ultimate tensile stress of 22.2% which varies from 279.98 ± 14.06 to 217.80 ± 3.23 MPa after β sterilization.

### Stress-strain curve—Mechanical behavior analysis

**Fig 3:**
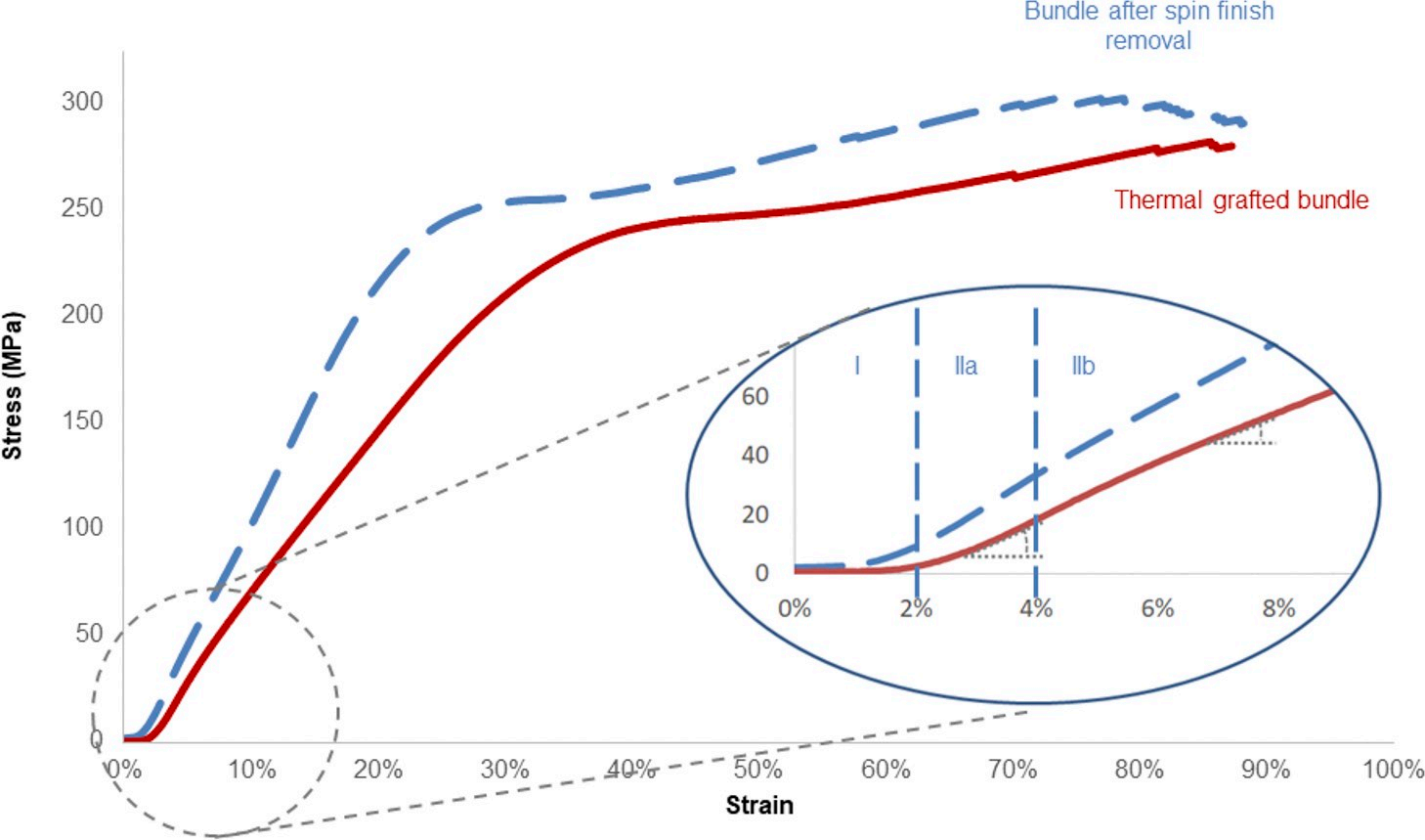
Typical strain-stress curves and delimitation of domains for bundles after spin finish removal (dashed line curve) and thermal grafted bundles (full line curve).

The stress-strain curves of the PCL fibers bundles present three domains (see Fig 3): (a) a first domain corresponding of the alignment of the fibers of the assessed bundle, (b) a second domain which corresponds to the elastic domain, followed by (c) a plastic domain going until the rupture of all the fibers. It is worth noting that the stress-strain curves obtained after the different chemical treatments of PCL bundles exhibit altered curves with two slopes within the elastic domain (Fig 3):

- a first linear elastic domain (IIa) corresponding to a deformation ε of 1.44 ± 0.23% of the samples and until a stress of 18.2 ± 3.7 MPa
- a second elastic (IIb) corresponding to a deformation ε of 15.5 ± 2.5% of the samples and a strain force of 150.5 ± 15 MPa. This non-linear elastic domain is present on the stress strain curves of all the PCL fibers bundles samples whatever the applied physico-chemical treatment. This is amplified in the case of the thermal grafting treatment which induces an approximate 25% decrease of the slope.
- a plastic domain (III) appearing for ε deformation of 81.8 ± 7.2% of sample deformation and for a stress of 286 ±34 MPa.

### Load-unload experiments

**Fig 4:**
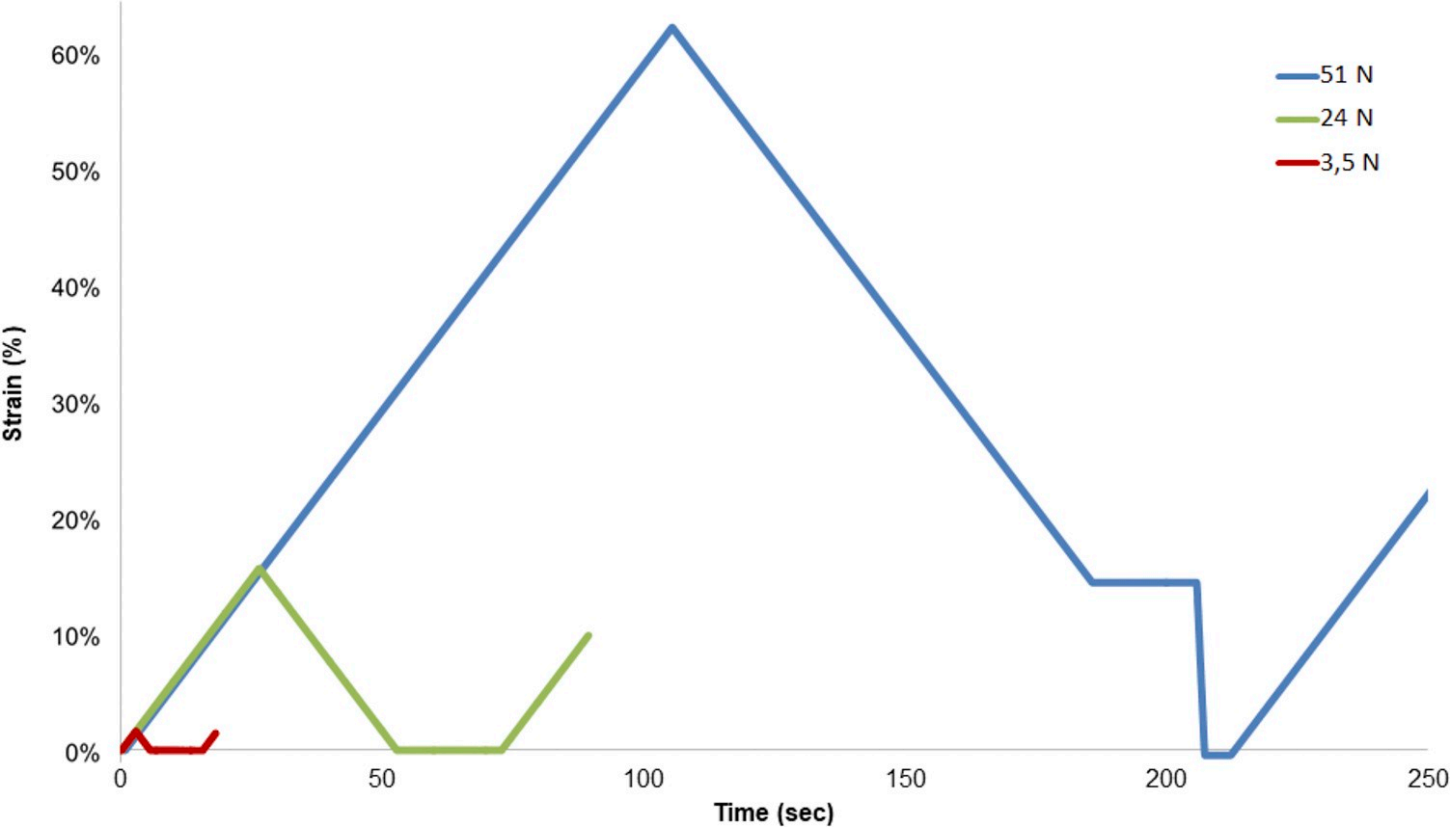
Deformation in time of bundles which endured loading of either 3.5N, 24N or 51N.

In order to define the elastic and the plastic domain limits, fibers bundle samples were submitted to load at 3.5N, 24N or 51N, corresponding to the required forces to respectively reach the domains IIa, IIb and III. The deformation against time chart shows that fibers bundles recover their initial stage without residual deformation for loading values 3.5N and 24N - elastic domain, whereas at 51N loading, a plateau value is observed corresponding to irreversible deformation of the fibers bundle samples (plastic domain) as seen in Fig 4.

**Fig 5:**
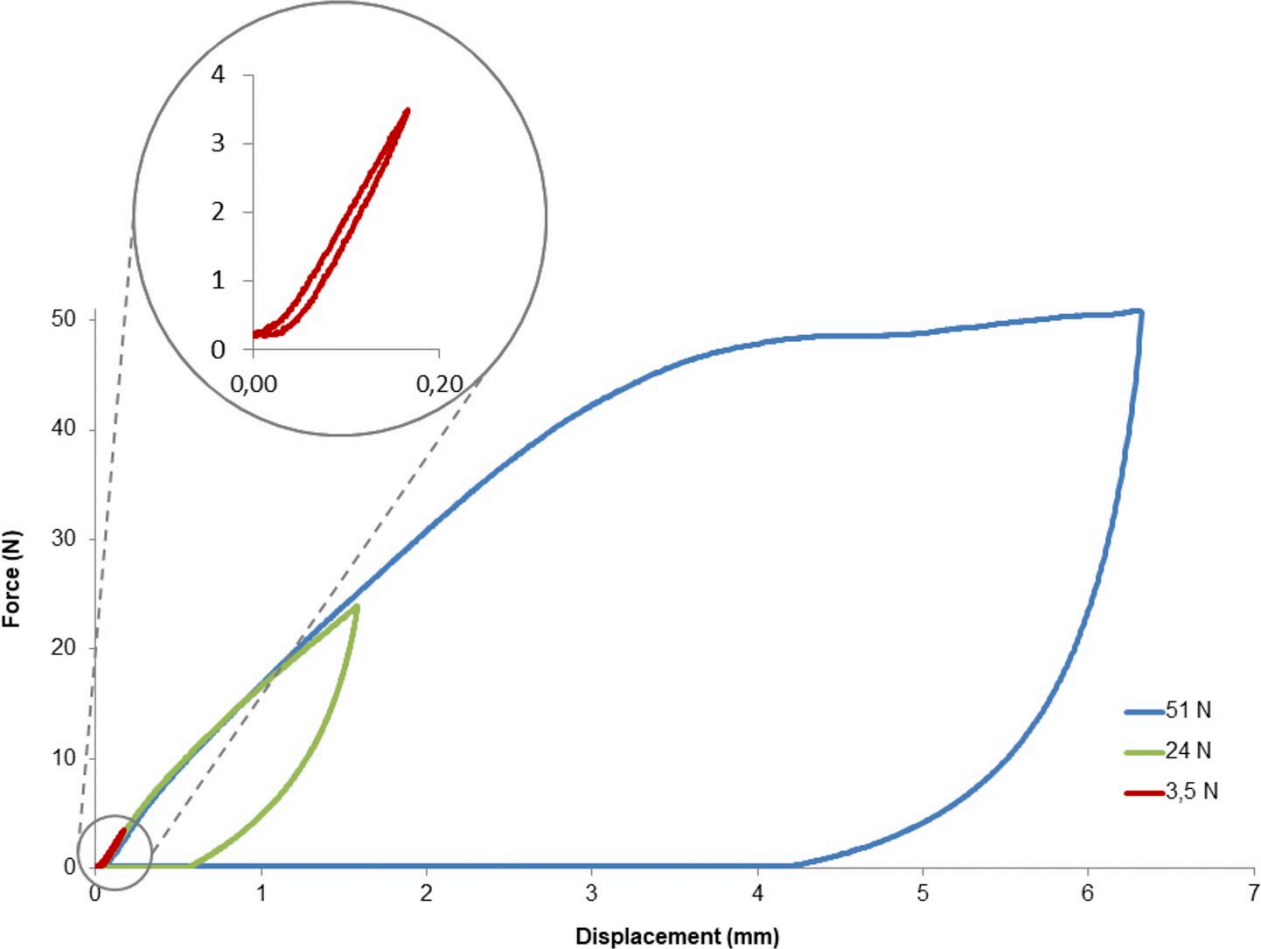
Hysteresis loops obtained after load-unload experiments onto grafted samples.

Moreover, from the same experiments, the force-displacement curves show an hysteresis loop for each of the three domains (Fig 5).

**Fig 6:**
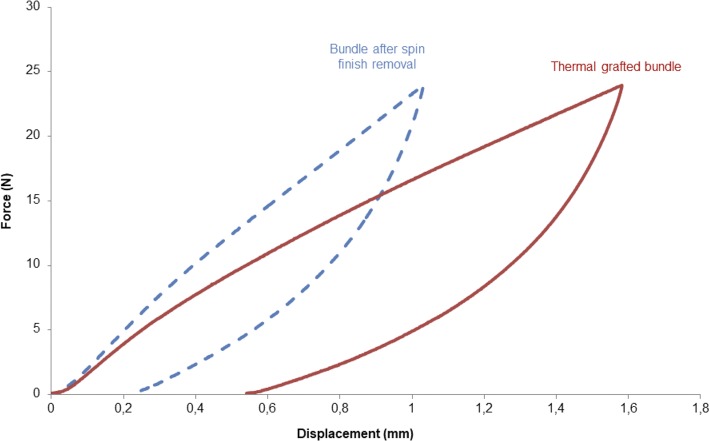
Comparaison of the hysteresis loops for ungrafted (dashed line curve) and grafted (full line curve) samples after a load of 24N.

Insofar as this is the non-linear elastic domain which is increased when the samples have undergone with thermal grafting, the energy loss was calculated from the hysteresis at 24N (Fig 6). For a non-grafted sample, the energy dissipation equals 6.5 ± 0.1 mJ whereas the dissipation of energy is doubled—equals 12.0 ± 0.9 mJ for pNaSS grafted samples.

### Microstructure of PCL

**Fig 7:**
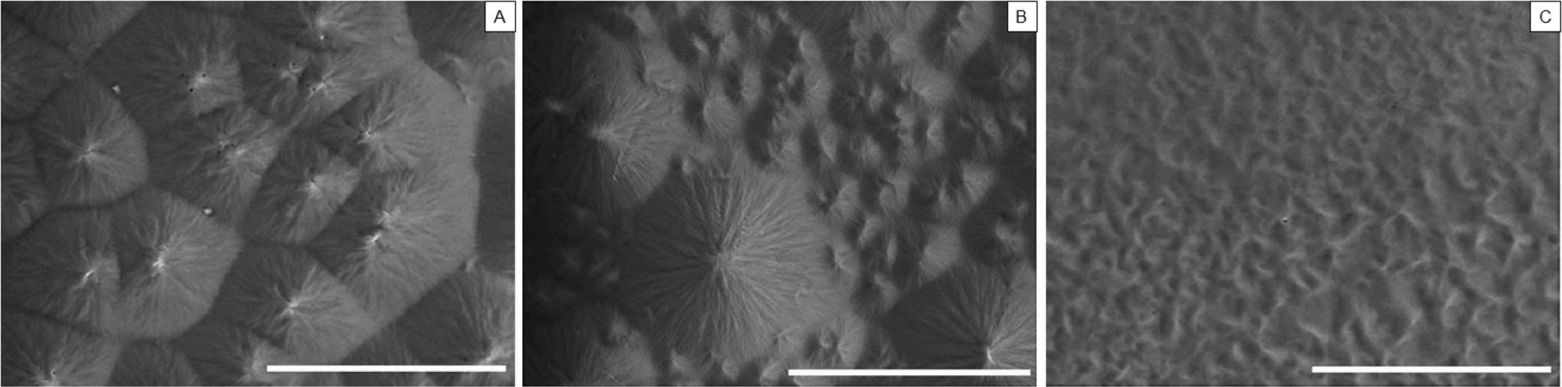
**SEM images of spin-coating PCL films after 1 night at room temperature (A); after 4 days at 45°C (B); after quenching (C)** (Scale bar = 50μm).

As the pNaSS grafting process of PCL requires working temperatures from room temperature to 45°C which is not so far from PCL Tm, the alteration of PCL microstructure has been observed by SEM images after different thermal treatments (Fig 7). PCL films left at room temperature after the spin coating process present a crystalline structure with spherulites of average size equaling 22.4 ± 6.1 μm separated by very straight lines. After 4 days at 45°C, the microstructure is changed and a rearrangement of the macromolecular chains within the crystalline domain was observed resulting in the formation of two types of spherulites: big spherulites of size average 27.9 ± 6.4 μm surrounded by smaller ones of 8.1 ± 2.4 μm size. When the film is quenched after melting an amorphous and homogeneous structure is observable.

## Discussion

### Cell response

The *in vitro* cell morphology study showed that the presence of a bioactive coating on the PCL fibers material like the pNaSS grafting significantly improved the cell spreading and the numbers of adhered cells when compared to virgin PCL fibers samples. This result was in agreement with the one observed on pNaSS grafted PCL films[36] and confirmed the interest of grafting pNaSS on PCL fibers bundles dedicated to the elaboration of implan synthetic ligament prosthesis. Indeed, it is well known that adhesion, spreading, proliferation and gene expression are interconnected key factors of a good cell activity[39]. The implantation of synthetic materials having a huge impact on the host response, the expected better cell response is encouraging for improving the host response. Nevertheless, the improvement of the biological response by the pNaSS grafting must not be done to the detriment of the mechanical properties especially for orthopedic applications and of the integrity of the structure of the material. It is worth noting that the stress-strain curves obtained for the PCL fibers bundles were comparable to that of native anterior cruciate ligaments.

### Mechanical behavior analysis

The mechanical stress-strain curves of PCL fibers bundles (see Fig 3) corresponded perfectly to a semi-crystalline material with a viscoelastic behavior [34]. Because of the values of its glass temperature Tg (-60°C) and melting point Tm (59 to 64°C), poly-caprolactone naturally behaves in caoutchoutic state at room temperature which allows mobility of the molecular chains when compared to other biodegradable polyesters as PLA and PGA[30,40,41].

The study of the mechanical properties of pNaSS grafted and ungrafted PCL fibers bundles demonstrated that a treatment like the removal of the spin finish, did not affect the material whereas others treatments as ozonation, radical grafting or β sterilization can influence the mechanical behavior of the final construct. Indeed, the results showed that the use of ionizing radiation like UV or β-rays increases the stiffness of the material but also its fragility resulting in a hardening of the material. The method of sterilization for the future prosthesis has to be chosen very carefully[42] and it will be necessary to extend this study to other sterilization processes as ethanol or ethylene oxide.

In contrast, other treatments like thermal radical grafting were found to increase the non-linear elasticity of the polymer, acting like an annealing step[43]. The first objective of the heating is the modification of the chemical surface by covalent binding of a bioactive polymer, but the results showed that it could also significantly increase the elasticity of the fibers until 18.8 ± 1.3%. In the case of the specific application of the ACL reconstruction, the need of a construct which can endure a reversible deformation around 13 ± 2% (average strain of the ACL during walking)[44] is strongly required to maintain the stability of the knee by avoiding a distension of the prosthesis. To summarize these data demonstrate, the possibility of improving the biocompatibility of the fibers surface and their elasticity in the same time during the same very efficient process step.

Moreover, we observed that the stiffness is decreasing with the thermal grafting, leading to a higher energy dissipation calculated onto the hysteresis loop. These results, added to the observation of SEM images done onto films after different thermal treatments, demonstrated the possibility of the PCL to modulate it microstructure with prior thermal treatments[45]. Taking into account that the knee temperature varies from 33°C at rest to 36.7°C after 1h walking[46], the importance of an orthopedic biomaterial implant which can modulate itself with temperature is given[45]. It could be interesting in further studies to mechanically evaluate PCL fibers bundles at temperatures varying from 33°C to 37°C in order to evaluate the evolution of their elasticity.

In a biological response control point of view, Abdel-Sayed et al. have demonstrated the possible impact of the dissipation energy of polymeric scaffolds onto the modulation of chondrogenic expression[47]. They observed a higher gene expression of the cells in contact with the material which has the closest energy dissipation to the healthy cartilage. With the help of the thermal grafting, we were able to increase the energy loss by our PCL fibers material, it will be interesting to study if we can modulate this parameter by modify the protocol of the grafting and then to study the gene expression of the osteoblast and the fibroblast in order to discover if we have the same effect for these type of cells.

## Conclusion

The aim of the study was to demonstrate that bioactive poly(caprolactone) fibers bundles can present similar mechanical properties when compared to native ACL. This was demonstrated by different mechanical assays and confirms the interest of the choice of PCL for the elaboration of ligament prosthesis with appropriate mechanical behavior. Moreover, even if PCL is a well-known biocompatible polymer, the functionalization of its surface by a grafted bioactive polymer can significantly improve the biological response to this polymer material. This evolution to an improvement of the biocompatibility requires taking the mechanic modulations of the raw material in account. By studying the mechanical properties after different chemical and physical treatments, we demonstrated the possibility to modulate these features, especially the elasticity, according to the grafting process used. Finally, because the anterior cruciate ligament is naturally stressed in cycle fatigue, fatigue assays need to be performed; these results will allow us to go on starting bioreactor studies.

## References

[1] Kaeding CC, Léger-St-Jean B, Magnussen RA. Epidemiology and Diagnosis of Anterior Cruciate Ligament Injuries. Clin Sports Med. 2017;36: 1–8. 10.1016/j.csm.2016.08.001 27871652

[2] Suart WM, Patrick MA, Betts HJ, Murray A, Pope JA. Rehabilitation following Dacron Graft Reconstruction of the Anterior Cruciate Ligament of the Knee. Physiotherapy. 1988;74: 528–530. 10.1016/S0031-9406(10)63401-3

[3] Klein W, Jensen K-U. Synovitis and artificial ligaments. Arthrosc J Arthrosc Relat Surg. 1992;8: 116–124. 10.1016/0749-8063(92)90145-2 1532312

[4] Thomson L, Houlton J, Allen M, Rushton N. Replacement of the anterior cruciate ligament with a coated carbon fibre prosthesis: a biomechanical study in goats. The Knee. 1994;1: 139–145. 10.1016/0968-0160(94)90028-0

[5] Kalaci A, Uruc V, Özden R, Duman IG, Dogramaci Y, Karapinar S, et al The Biocompatibility of Nitinol in Knee Joint Spaces and Femoral Tunnels: An Experimental Study in Rats. J Hard Tissue Biol. 2014;23: 317–322.

[6] Mowbray MAS, McLeod ARM, Barry M, Cooke WD, O’Brien TK. Early failure in an artificial anterior cruciate ligament scaffold. The Knee. 1997;4: 35–40. 10.1016/S0968-0160(96)00225-6

[7] Guidoin M-F, Marois Y, Bejui J, Poddevin N, King MW, Guidoin R. Analysis of retrieved polymer fiber based replacements for the ACL. Biomaterials. 2000;21: 2461–2474. 10.1016/S0142-9612(00)00114-9 11055294

[8] Marois Y, Roy R, Vidovszky T, King MW, Bélanger AY, Chaput C, et al Histopathological and immunological investigations of synthetic fibres and structures used in three prosthetic anterior cruciate ligaments: in vivo study in the rat. Biomaterials. 1993;14: 255–262. 8476995 10.1016/0142-9612(93)90115-i

[9] Iannace S, Sabatini G, Ambrosio L, Nicolais L. Mechanical behaviour of composite artificial tendons and ligaments. Biomaterials. 1995;16: 675–680. 10.1016/0142-9612(95)99693-G 7578769

[10] Seitz H, Marlovits S, Schwendenwein I, Müller E, Vécsei V. Biocompatibility of polyethylene terephthalate (Trevira hochfest) augmentation device in repair of the anterior cruciate ligament. Biomaterials. 1998;19: 189–196. 10.1016/S0142-9612(97)00201-9 9678867

[11] Viateau V, Manassero M, Anagnostou F, Guérard S, Mitton D, Migonney V. Biological and Biomechanical Evaluation of the Ligament Advanced Reinforcement System (LARS AC) in a Sheep Model of Anterior Cruciate Ligament Replacement: A 3-Month and 12-Month Study. Arthrosc J Arthrosc Relat Surg. 2013;29: 1079–1088. 10.1016/j.arthro.2013.02.025 23726110

[12] Kew SJ, Gwynne JH, Enea D, Abu-Rub M, Pandit A, Zeugolis D, et al Regeneration and repair of tendon and ligament tissue using collagen fibre biomaterials. Acta Biomater. 2011;7: 3237–3247. 10.1016/j.actbio.2011.06.002 21689792

[13] Freeman JW, Woods MD, Laurencin CT. Tissue engineering of the anterior cruciate ligament using a braid–twist scaffold design. J Biomech. 2007;40: 2029–2036. 10.1016/j.jbiomech.2006.09.025 17097666 PMC2034317

[14] Rawal A, Sibal A, Saraswat H, Quddus Khan S. Tensile behaviour of structurally gradient braided prostheses for anterior cruciate ligaments. J Mech Behav Biomed Mater. 2016;54: 305–315. 10.1016/j.jmbbm.2015.09.018 26505530

[15] Pauly HM, Kelly DJ, Popat KC, Trujillo NA, Dunne NJ, McCarthy HO, et al Mechanical properties and cellular response of novel electrospun nanofibers for ligament tissue engineering: Effects of orientation and geometry. J Mech Behav Biomed Mater. 2016;61: 258–270. 10.1016/j.jmbbm.2016.03.022 27082129

[16] Mollica F, Ventre M, Sarracino F, Ambrosio L, Nicolais L. Mechanical properties and modelling of a hydrophilic composite used as a biomaterial. Compos Sci Technol. 2006;66: 92–101. 10.1016/j.compscitech.2005.05.022

[17] Li H, Chen C, Zhang S, Jiang J, Tao H, Xu J, et al The use of layer by layer self-assembled coatings of hyaluronic acid and cationized gelatin to improve the biocompatibility of poly(ethylene terephthalate) artificial ligaments for reconstruction of the anterior cruciate ligament. Acta Biomater. 2012;8: 4007–4019. 10.1016/j.actbio.2012.07.008 22813848

[18] Deepthi S, Jeevitha K, Nivedhitha Sundaram M, Chennazhi KP, Jayakumar R. Chitosan–hyaluronic acid hydrogel coated poly(caprolactone) multiscale bilayer scaffold for ligament regeneration. Chem Eng J. 2015;260: 478–485. 10.1016/j.cej.2014.08.106

[19] Miculescu F, Maidaniuc A, Voicu SI, Thakur VK, Stan GE, Ciocan LT. Progress in Hydroxyapatite–Starch Based Sustainable Biomaterials for Biomedical Bone Substitution Applications. ACS Sustain Chem Eng. 2017;5: 8491–8512. 10.1021/acssuschemeng.7b02314

[20] Ratner BD, Hoffman AS, Schoen FJ, Lemons JE. Biomaterials Science: An Introduction to Materials in Medicine Academic Press; 2004.

[21] Laurencin CT, Freeman JW. Ligament tissue engineering: An evolutionary materials science approach. Biomaterials. 2005;26: 7530–7536. 10.1016/j.biomaterials.2005.05.073 16045982

[22] Cooper JA, Sahota JS, Gorum WJ, Carter J, Doty SB, Laurencin CT. Biomimetic tissue-engineered anterior cruciate ligament replacement. Proc Natl Acad Sci. 2007;104: 3049–3054. 10.1073/pnas.0608837104 17360607 PMC1805619

[23] Murray MM, Flutie BM, Kalish LA, Ecklund K, Fleming BC, Proffen BL, et al The Bridge-Enhanced Anterior Cruciate Ligament Repair (BEAR) Procedure: An Early Feasibility Cohort Study. Orthop J Sports Med. 2016;4: 232596711667217. 10.1177/2325967116672176 27900338 PMC5120682

[24] Belleney J, Hélary G, Migonney V. Terpolymerization of methyl methacrylate, poly(ethylene glycol) methyl ether methacrylate or poly(ethylene glycol) ethyl ether methacrylate with methacrylic acid and sodium styrene sulfonate: determination of the reactivity ratios. Eur Polym J. 2002;38: 439–444. 10.1016/S0014-3057(01)00205-1

[25] Felgueiras H, Migonney V. Sulfonate groups grafted on Ti6Al4V favor MC3T3-E1 cell performance in serum free medium conditions. Mater Sci Eng C. 2014;39: 196–202. 10.1016/j.msec.2014.03.013 24863216

[26] Pavon-Djavid G, Gamble LJ, Ciobanu M, Gueguen V, Castner DG, Migonney V. Bioactive Poly(ethylene terephthalate) Fibers and Fabrics: Grafting, Chemical Characterization, and Biological Assessment. Biomacromolecules. 2007;8: 3317–3325. 10.1021/bm070344i 17929865

[27] Brulez B, Laboureau J-P, Migonney V, Ciobanu M, Pavon-Djavid G, Siove A. Biomimetic Prosthetic Ligament and Production Method Thereof [Internet]. WO2004067051 (A1), 2004.

[28] Vaquette C, Viateau V, Guérard S, Anagnostou F, Manassero M, Castner DG, et al The effect of polystyrene sodium sulfonate grafting on polyethylene terephthalate artificial ligaments on in vitro mineralisation and in vivo bone tissue integration. Biomaterials. 2013;34: 7048–7063. 10.1016/j.biomaterials.2013.05.058 23790438 PMC3779617

[29] Viateau V, Zhou J, Guérard S, Manassero M, Thourot M, Anagnostou F, et al Ligart: ligament synthétique «bioactif» et «biointégrable» permettant la réhabilitation rapide du patient: greffage chimique, évaluations biologiques in vivo, expérimentation animale, étude préclinique. IRBM. 2011;32: 118–122. 10.1016/j.irbm.2011.01.007

[30] Woodruff MA, Hutmacher DW. The return of a forgotten polymer—Polycaprolactone in the 21st century. Prog Polym Sci. 2010;35: 1217–1256. 10.1016/j.progpolymsci.2010.04.002

[31] Pitt CG, Chasalow FI, Hibionada YM, Klimas DM, Schindler A. Aliphatic polyesters. I. The degradation of poly (ϵ-caprolactone) in vivo. J Appl Polym Sci. 1981;26: 3779–3787.

[32] Sun H, Mei L, Song C, Cui X, Wang P. The in vivo degradation, absorption and excretion of PCL-based implant. Biomaterials. 2006;27: 1735–1740. 10.1016/j.biomaterials.2005.09.019 16198413

[33] Höglund A, Hakkarainen M, Albertsson A. Degradation Profile of Poly(ϵ‐caprolactone)–the Influence of Macroscopic and Macromolecular Biomaterial Design. J Macromol Sci Part A. 2007;44: 1041–1046. 10.1080/10601320701424487

[34] Noroozi N, Thomson JA, Noroozi N, Schafer LL, Hatzikiriakos SG. Viscoelastic behaviour and flow instabilities of biodegradable poly (ε-caprolactone) polyesters. Rheol Acta. 2012;51: 179–192. 10.1007/s00397-011-0586-6

[35] Djaker N, Brustlein S, Rohman G, Huot S, de la Chapelle ML, Migonney V. Characterization of a synthetic bioactive polymer by nonlinear optical microscopy. Biomed Opt Express. 2014;5: 149 10.1364/BOE.5.000149 24466483 PMC3891327

[36] Rohman G, Huot S, Vilas-Boas M, Radu-Bostan G, Castner DG, Migonney V. The grafting of a thin layer of poly(sodium styrene sulfonate) onto poly(ε-caprolactone) surface can enhance fibroblast behavior. J Mater Sci Mater Med. 2015;26 10.1007/s10856-015-5635-8 26155977 PMC4554533

[37] Huang Y-P, Chen T-K. Effect of surface treatment on pet spinning and the yarn property. Colloids Surf Physicochem Eng Asp. 2007;295: 75–80. 10.1016/j.colsurfa.2006.08.034

[38] Ciobanu M, Siove A, Gueguen V, Gamble LJ, Castner DG, Migonney V. Radical graft polymerization of styrene sulfonate on poly (ethylene terephthalate) films for ACL applications:“grafting from” and chemical characterization. Biomacromolecules. 2006;7: 755–760. 10.1021/bm050694+ 16529411

[39] Haddad O, Gumez L, Hawse JR, Subramaniam M, Spelsberg TC, Bensamoun SF. TIEG1-null tenocytes display age-dependent differences in their gene expression, adhesion, spreading and proliferation properties. Exp Cell Res. 2011;317: 1726–1735. 10.1016/j.yexcr.2011.05.007 21620830 PMC3215103

[40] Van Dommelen J van, Parks DM, Boyce MC, Brekelmans WAM, Baaijens FPT. Micromechanical modeling of the elasto-viscoplastic behavior of semi-crystalline polymers. J Mech Phys Solids. 2003;51: 519–541.

[41] Uchida M, Tada N. Micro-, meso- to macroscopic modeling of deformation behavior of semi-crystalline polymer. Int J Plast. 2013;49: 164–184. 10.1016/j.ijplas.2013.03.007

[42] Proffen BL, Perrone GS, Fleming BC, Sieker JT, Kramer J, Hawes ML, et al Electron Beam Sterilization Does Not Have a Detrimental Effect on the Ability of Extracellular Matrix Scaffolds to Support In Vivo Ligament Healing. J Orthop Res Off Publ Orthop Res Soc. 2015;33: 1015–1023. 10.1002/jor.22855 25676876 PMC4517185

[43] Farah S, Anderson DG, Langer R. Physical and mechanical properties of PLA, and their functions in widespread applications—A comprehensive review. Adv Drug Deliv Rev. 2016;107: 367–392. 10.1016/j.addr.2016.06.012 27356150

[44] Taylor KA, Cutcliffe HC, Queen RM, Utturkar GM, Spritzer CE, Garrett WE, et al In vivo measurement of ACL length and relative strain during walking. J Biomech. 2013;46: 478–483. 10.1016/j.jbiomech.2012.10.031 23178040 PMC3552116

[45] Nelson MT, Pattanaik L, Allen M, Gerbich M, Hux K, Allen M, et al Recrystallization improves the mechanical properties of sintered electrospun polycaprolactone. J Mech Behav Biomed Mater. 2014;30: 150–158. 10.1016/j.jmbbm.2013.11.004 24295966

[46] Abdel-Sayed P, Moghadam MN, Salomir R, Tchernin D, Pioletti DP. Intrinsic viscoelasticity increases temperature in knee cartilage under physiological loading. J Mech Behav Biomed Mater. 2014;30: 123–130. 10.1016/j.jmbbm.2013.10.025 24287306

[47] Abdel-Sayed P, Darwiche SE, Kettenberger U, Pioletti DP. The role of energy dissipation of polymeric scaffolds in the mechanobiological modulation of chondrogenic expression. Biomaterials. 2014;35: 1890–1897. 10.1016/j.biomaterials.2013.11.048 24331703

